# HIV Pol Inhibits HIV Budding and Mediates the Severe Budding Defect of Gag-Pol

**DOI:** 10.1371/journal.pone.0029421

**Published:** 2012-01-03

**Authors:** Xin Gan, Stephen J. Gould

**Affiliations:** Department of Biological Chemistry, The Johns Hopkins University School of Medicine, Baltimore, Maryland, United States of America; University of Rochester, United States of America

## Abstract

The prevailing hypothesis of HIV budding posits that the viral Gag protein drives budding, and that the Gag p6 peptide plays an essential role by recruiting host-cell budding factors to sites of HIV assembly. HIV also expresses a second Gag protein, p160 Gag-Pol, which lacks p6 and fails to bud from cells, consistent with the prevailing hypothesis of HIV budding. However, we show here that the severe budding defect of Gag-Pol is not caused by the absence of p6, but rather, by the presence of Pol. Specifically, we show that (*i*) the budding defect of Gag-Pol is unaffected by loss of HIV protease activity and is therefore an intrinsic property of the Gag-Pol polyprotein, (*ii*) the N-terminal 433 amino acids of Gag and Gag-Pol are sufficient to drive virus budding even though they lack p6, (*iii*) the severe budding defect of Gag-Pol is caused by a dominant, *cis*-acting inhibitor of budding in the HIV Pol domain, and (*iv*) Gag-Pol inhibits Gag and virus budding in *trans*, even at normal levels of Gag and Gag-Pol expression. These and other data support an alternative hypothesis of HIV budding as a process that is mediated by the normal, non-viral pathway of exosome/microvesicle biogenesis.

## Introduction

Retrovirus budding is an important but incompletely understood process, with relevance to both the viral lifecycle [1], [2], [3] and the biogenesis of secreted vesicles (e.g. exosomes and microvesicles (EMVs) [4], [5]). Early studies on HIV budding demonstrated that loss of the Gag p6 domain caused a severe defect in virus budding [6], and that mutation of short peptide motifs within p6 could also cause a similar phenotype [7]. These motifs (PTAP, YPxL) bind directly to components of the *endosomal sorting complexes required for transport* (ESCRT) machinery, which is required for cytokinesis, biogenesis of multivesicular bodies, autophagy, and also for HIV budding [8], [9], [10]. Based on these results, it has been proposed that the p6 domain represents the primary budding information in HIV [1], [2]. This hypothesis is also consistent with the current model of outward vesicle budding (outward = away from the cytoplasm), which posits a central role for the ESCRT machinery in cargo selection and vesicle budding [5], [11], [12].

This hypothesis of HIV budding is consistent with some lines of evidence but difficult to reconcile with others. In particular, Freed and others observed that p6-deficient HIV shows ‘*no defect in particle release*’ from human T-cells [13], [14], undermining the idea that p6 plays a critical role in budding. More recently, we established that plasma membrane (PM)-binding and higher-order oligomerization are the primary budding signals in HIV Gag, and are located in its matrix (MA), capsid (CA), and nucleocapsid (NC) domains [14], [15]. As for the severe budding defect seen for p6-deficient and PTAP-deficient forms of HIV in 293T and certain other cell types [6], [13], this appears to be caused by the activation of an inhibitory budding signal (IBS) in the SP2 domain of Gag [15], rather than the loss of positive budding information. Taken together, these observations support an alternative hypothesis in which retroviruses bud by the normal, non-viral pathway of EMV biogenesis [4].

EMVs are small, membrane-bound vesicles secreted by a wide array of animal cells and mediate the release of specific subsets of proteins, lipids, carbohydrates, and nucleic acids [16], [17]. The EMV-based hypothesis of HIV budding is also supported by (i) similarities between the host-cell proteins, lipids, and carbohydrates that are present on HIV particles and on EMVs [4], [18], [19], (ii) the fact that EMVs and HIV particles bud from the same locations of human T-cells, macrophages, and polarized leukocytes [14], [19], [20], [21], [22], [23], [24], and (iii) the observation that PM-binding and higher-order oligomerization target diverse proteins to both HIV particles and EMVs [14], [15], [22].

Although the existing data favor an EMV-based hypothesis of HIV budding, it is unclear whether this model can explain the severe budding defect reported for the HIV Gag-Pol protein [25], [26], [27]. HIV expresses Gag and Gag-Pol from the same mRNA. Both proteins share the identical N-terminal 433 amino acids but differ at their C-terminus. In p55 Gag, the shared N-terminal 433 amino acids is followed by the 16 amino acid-long spacer peptide 2 (SP2) and the 52 amino acid-long p6 peptide. In p160 Gag-Pol, which lacks SP2 and p6, the shared N-terminal 433 amino acids are instead followed by the 1003 amino acid-long Pol domain (p160 Gag-Pol is generated by a −1 ribosomal frameshift at codon 433 of the Gag ORF). Under the prevailing hypothesis of HIV budding, Gag should bud from cells and Gag-Pol should not, because the former protein possesses p6 and the latter does not. However, we show here that the severe budding defect of Gag-Pol is caused by the presence of a dominant, *cis*-acting inhibitor of budding in HIV Pol, and not by the absence of p6. Furthermore, we show that the N-terminal 433 amino acids of HIV Gag-Pol are sufficient to bud from cells, supporting the hypothesis that HIV budding is mediated by the EMV biogenesis pathway.

## Results

### Protease does not mediate the budding defect of HIV Gag-Pol

**Figure 1:**
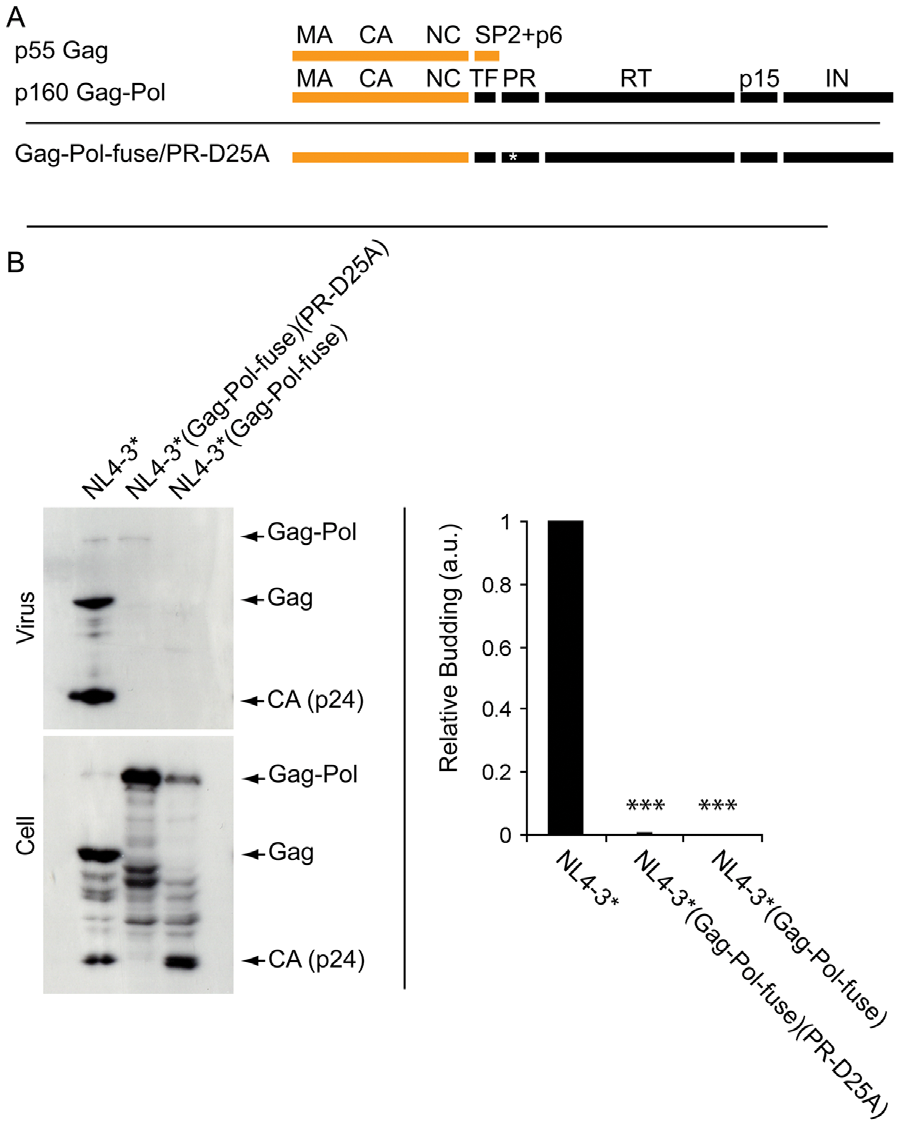
Gag-Pol does not bud from 293T cells. (**A**) Line diagram of the p55 HIV Gag and p160 HIV Gag-Pol proteins, as well as the protein product of the NL4-3*/Gag-Pol^fuse^/PR^D25A^ provirus. The full-length p55 Gag and p160 Gag-Pol proteins contain a shared N-terminal 433 amino acids containing the MA, CA, and NC domains but different C-terminal extensions, SP2 and p6 in the case of Gag, and Pol in the case of Gag-Pol. (**B**) Anti-Gag immunoblots of cell and virus lysates generated from 293T cells transfected with the proviruses NL4-3*, NL4-3*/Gag-Pol^fuse^/PR^D25A^ and NL4-3*/Gag-Pol^fuse^. Bar graph shows the average+/−1 standard deviation from three trials. The three stars refers to a p value of less than 0.0005.

HIV expresses its Gag and Gag-Pol proteins from a single mRNA, with Gag being the primary translation product and Gag-Pol being generated by a −1 ribosomal frameshift at codon 433 of the Gag ORF (**Fig. 1A**). As a result, Gag and Gag-Pol share the same N-terminal 433 amino acids (the MA, CA, and NC domains) but have different C-terminal domains. In Gag, this divergent sequence consists of the SP2 and p6 peptides, while in Gag-Pol it corresponds to the 1003 amino acids of Pol, itself comprised of transframe (TF), protease (PR), reverse transcriptase (RT), p15, and integrase (IN) domains [28].

It has been established that fusing the Gag and Pol reading frames in an HIV provirus (at the site of the Gag-Pol translational frameshift) results in much higher levels of Gag-Pol expression, prevents the synthesis of Gag, and causes a severe defect in HIV budding [25], [26], [27]. Elevated expression of HIV Gag-Pol also leads to elevated expression of PR activity, a known inhibitor of HIV budding, as well as to the extensive cleavage of cell-associated Gag-Pol. This raises the possibility that the budding defect observed for Gag-Pol virus reflects the elevated, unregulated, and/or premature PR activity, rather than the intrinsic budding activity of the full-length p160 Gag-Pol protein [25], [26], [27]. To assess the role of PR activity in the budding defect of Gag-Pol virus we compared the budding of control HIV (NL4-3*, our designation [15] for NL4-3-ΔE-GFP, an ENV-deficient form of NL4-3 used for quantitative assays of HIV function [29]) to that of a derivative in which the Gag and Pol reading frames were fused (NL4-3*/Gag-Pol^fuse^), and also to that of a matched provirus in which PR was mutationally inactivated (NL4-3*/Gag-Pol^fuse^/PR^D25A^) [7]. More specifically, 293T cells were transfected with the HIV proviruses NL4-3*, NL4-3*/Gag-Pol^fuse^, and NL4-3*/Gag-Pol^fuse^/PR^D25A^, incubated for two days, and then cells and virus particles were collected (viruses were purified by differential centrifugation from the tissue culture supernantant). Cell and virus samples were then lysed in SDS-PAGE sample buffer and the lysates were processed for immunoblot using antibodies specific for the CA domain of Gag-Pol (**Fig. 1**). These virus budding experiments revealed that NL4-3* budded efficiently from 293T cells, as expected. However, we detected very little budding for either NL4-3*/Gag-Pol^fuse^ (0% of control; n = 3) or NL4-3*/Gag-Pol^fuse^/PR^D25A^ (0.2+/−0.3% of control; n = 3; p = 4.3×10^−6^) (**Fig. 1A,B**). These results confirm that the Gag-Pol protein has a severe defect in budding, and also demonstrate that the HIV protease, PR, does not cause the budding defect of Gag-Pol.

### The budding defect of Gag-Pol is not caused by its lack of p6 sequences

**Figure 2:**
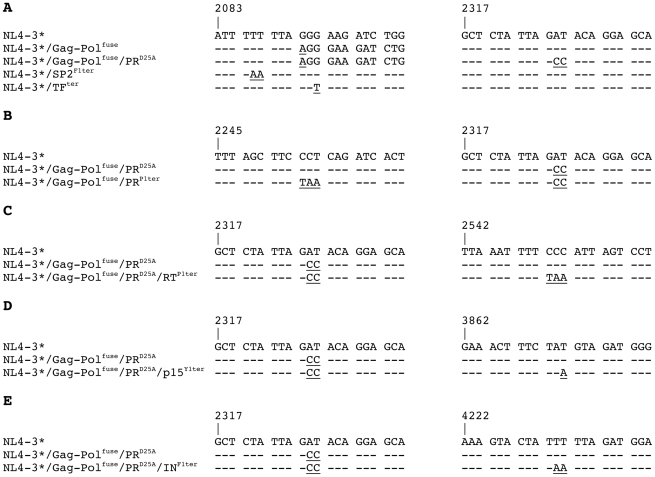
Relevant DNA sequences of control and mutant HIV proviruses. (**A**) Short segments of the NL4-3* DNA sequence and their alteration in the proviruses NL4-3*/Gag-Pol^fuse^, NL4-3*/Gag-Pol^fuse^/PR^D25A^, NL4-3*/SP2^F1ter^, and NL4-3*/TF^ter^, respectively. (**B**) DNA sequence fragments of NL4-3* in the vicinity of the PR^D25A^ and the PR^P1ter^ mutations in NL4-3*/Gag-Pol^fuse^/PR^D25A^ and NL4-3*/Gag-Pol^fuse^/PR^P1ter^, respectivel. (**C**) DNA sequence of the PR^D25A^ and the RT^P1ter^ mutations in NL4-3*/Gag-Pol^fuse^/PR^D25A^ and NL4-3*/Gag-Pol^fuse^/PR^D25A^/RT^P1ter^. (D) DNA sequence alignments in the vicinity of the PR^D25A^ and the p15^Y1ter^ mutations in NL4-3*/Gag-Pol^fuse^/PR^D25A^ and NL4-3*/Gag-Pol^fuse^/PR^D25A^/p15^Y1ter^. (E) DNA sequences surrounding the PR^D25A^ and the IN^F1ter^ mutations in NL4-3*/Gag-Pol^fuse^/PR^D25A^ and NL4-3*/Gag-Pol^fuse^/PR^D25A^/IN^F1ter^.

To test whether the budding defect of p160 Gag-Pol is due to the absence of p6 sequences or whether it might be caused by some other difference between it and p55 Gag, we followed the budding of another HIV mutant, NL4-3*/SP2^F1ter^. This virus replaces the first codon of the SP2 domain (Phe1) with a nonsense mutation, expresses only the N-terminal 433 amino acids that are shared by the Gag and Gag-Pol proteins, and lacks the SP2 and p6 domains of p55 Gag and the Pol domain of p160 Gag-Pol. Moreover, we simplified the analysis of its budding by comparing the release of NL4-3*/SP2^F1ter^ to that of NL4-3*/TF^ter^, an HIV mutant that expresses only p55 Gag (Fig. 1A; the TF^ter^ mutation has a nonsense mutation in the Pol ORF just downstream of the ribosomal frameshift [28]), lacks PR activity, and therefore doesn't cleave Gag into smaller polypeptides. 293T cells were transfected with NL4-3*/TF^ter^, NL4-3*/Gag-Pol^fuse^/PR^D25A^, and NL4-3*/SP2^F1ter^, cell and virus lysates were collected, and these were processed for immunoblot using anti-CA antibody (**Figs. 2** **,** **3**). NL4-3*/SP2^F1ter^ budded quite well, 73+/−9% relative to NL4-3*/TF^ter^ (n = 3; p = 0.033). This was ∼70-fold more budding than seen for NL4-3*/Gag-Pol^fuse^/PR^D25A^, which budded at only 1.1+/−2% of control (n = 3; p = 1.2×10^−4^). These data demonstrate that the budding defect of Gag-Pol is caused by the presence of Pol sequences and not by the absence of p6 sequences.

**Figure 3:**
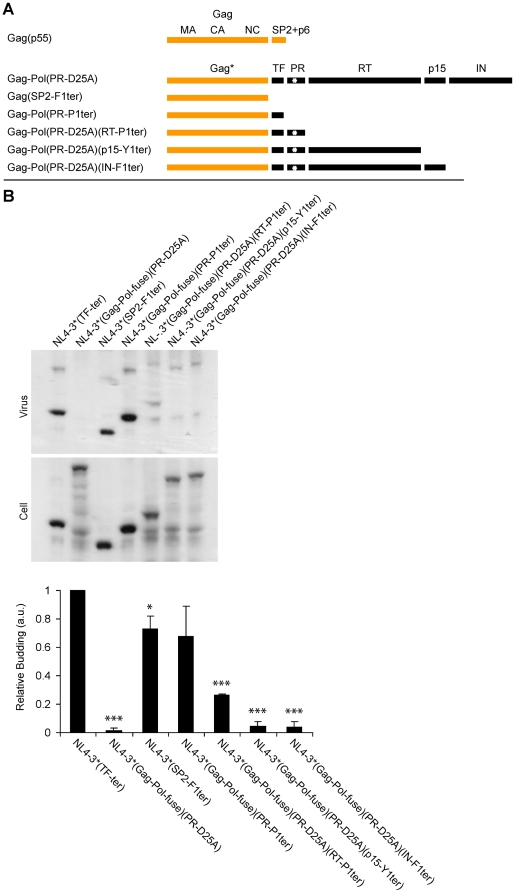
Relative budding of different C-terminal truncation mutations of Gag-Pol. (A) Line diagram of proteins expressed by the proviruses NL4-3*/TF^ter^ (Gag(p55)), NL4-3*/Gag-Pol^fuse^/PR^D25A^ (Gag-Pol(PR-D25A)), NL4-3*/SP2^F1ter^ (Gag(SP2-F1ter), NL4-3*/Gag-Pol^fuse^/PR^P1ter^ (Gag-Pol(PR-P1ter)), NL4-3*/Gag-Pol^fuse^/PR^D25A^/RT^P1ter^ (Gag-Pol(PR-D25A)(RT-P1ter)), NL4-3*/Gag-Pol^fuse^/PR^D25A^/p15^Y1ter^ (Gag-Pol(PR-D25A)(p15-Y1ter)), and NL4-3*/Gag-Pol^fuse^/PR^D25A^/IN^F1ter^ (Gag-Pol(PR-D25A)(IN-F1ter)), respectively. The upper line corresponds to the full-length p55 Gag protein, which contains p6, and is the only Gag-containing protein expressed by NL4-3*/TF^ter^. Gag* refers to the region of Gag shared by both Gag and Gag-Pol that lacks p6. (B) Anti-Gag immunoblots of cell and virus lysates generated from 293T cells transfected with the same proviruses. Bar graph shows the average +/−1 standard deviation from three trials. A single star refers to a p value of less than 0.05; three stars refers to a p value of less than 0.0005.

We next explored the regions of Pol that contribute to the budding defect of p160 Gag-Pol. For this, we generated derivatives of NL4-3*/Gag-Pol^fuse^ that contained stop codons immediately after the TF, PR, RT, and p15 domains of the Pol ORF (**Fig. 3A**). Following the expression of these proviruses in 293T cells, cell and EMV lysates were prepared and examined by immunoblot (**Fig. 3B**). NL4-3*/Gag-Pol^fuse^/PR^P1ter^, which expressed a Gag-Pol protein containing the first 433 amino acids of Gag and the TF domain of Pol, budded at 68+/−21% the level of NL4-3*/TF^ter^ (n = 3; p = 0.12). This is essentially the same level of budding that we observed for NL4-3*/SP2^F1ter^, which expresses just the MA-CA-SP1-NC domains of Gag. NL4-3*/Gag-Pol^fuse^/PR^D25A^/RT^P1ter^ is designed to express a longer Gag-Pol protein consisting of the MA, CA, NC, TF, and PR domains. It budded a bit less, 26+/−6% relative to NL4-3*/TF^ter^ (n = 3; p = 2.3×10^−5^). Inclusion of the RT domain caused an even larger decrease in HIV budding, to 4.3+/−3% (n = 3; p = 3.6×10^−4^) for NL4-3*/Gag-Pol^fuse^/PR^D25A^/p15^Y1ter^ and to 3.9+/−4% (n = 3; p = 5.1×10^−4^) for NL4-3*/Gag-Pol^fuse^/PR^D25A^/IN^F1ter^. These data demonstrate that the severe budding defect of Gag-Pol is caused primarily by sequences within the RT domain of Pol, and perhaps also by sequences within its PR domain.

### Gag-Pol inhibits HIV budding in *trans*

**Figure 4:**
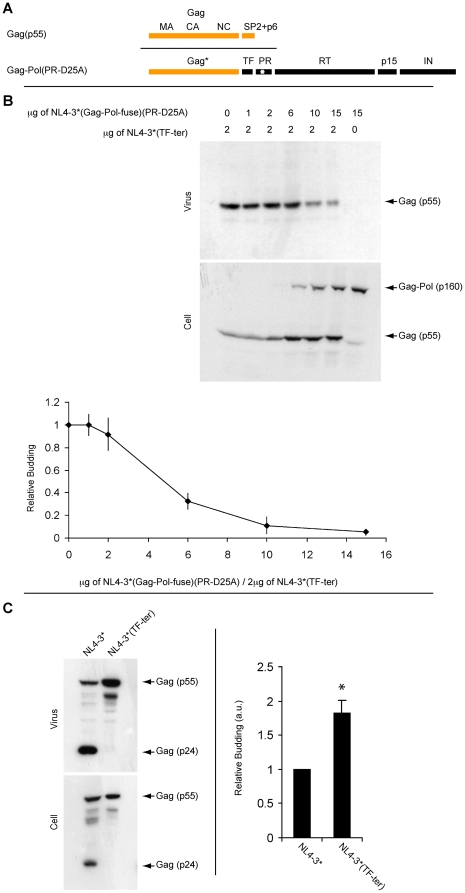
Expression of Gag-Pol inhibits HIV budding. (A) Line diagram of the Gag and Gag-Pol proteins expressed by NL4-3*/TF^ter^ and NL4-3*/Gag-Pol^fuse^/PR^D25A^, respectively. The upper line corresponds to the full-length p55 Gag protein, which contains p6, and is the only Gag-containing protein expressed by NL4-3*/TF^ter^. Gag* refers to the region of Gag shared by both Gag and Gag-Pol, a region that lacks p6. (B) Anti-Gag immunoblots of cell and virus lysates generated from 293T cells co-transfected with different amounts of the proviruses NL4-3*/TF^ter^ and NL4-3*/Gag-Pol^fuse^/PR^D25A^. The line graph shows the average +/−1 standard deviation from three separate trials. (C) Anti-Gag immunoblots of cell and virus lysates generated from 293T cells transfected with the proviruses NL4-3* and NL4-3*/TF^ter^. Bar graph shows the average +/−1 standard deviation from three trials. A single star refers to a p value of less than 0.05.

The inhibitory effect of Pol on Gag and HIV budding, coupled with the known co-assembly of Gag and Gag-Pol polyproteins [26], raises the possibility that Gag-Pol might also inhibit the budding of HIV in *trans*. To test this hypothesis we transfected 293T cells with a mix of (i) NL4-3*/TF^ter^, which expresses only Gag and no Gag-Pol, and (ii) NL4-3*/Gag-Pol^fuse^/PR^D25A^, which expresses Gag-Pol but no Gag (**Fig. 4A**). Two days later we collected cell and virus lysates and processed each by immunoblot using antibodies specific for the CA domain of the Gag and Gag-Pol proteins (**Fig. 4B**). These experiments revealed that expression of PR-deficient Gag-Pol had a dominant, inhibitory effect on HIV budding in *trans*. Peak inhibition, an ∼20-fold decrease (5.4+/−3%; n = 3; p = 2.7×10^−4^), was observed at the highest ratio of Gag-Pol∶Gag provirus, 7.5∶1. Significant inhibition of HIV budding was also observed at the lower Gag-Pol∶Gag ratios of 5∶1 (11+/−7%; n = 3; p = 2.3×10^−3^) and 3∶1 (32+/−7%; n = 3; p = 3.7×10^−3^). We failed to detect significant inhibition at Gag-Pol∶Gag ratios of 1∶1 (92+/−14%; n = 3; p = 0.42) or 1∶2 (99+/−9%; n = 3; p = 0.97).

### HIV Gag-Pol inhibits HIV budding

The inhibitory effect of Gag-Pol on HIV budding raised the possibility that Gag-Pol normally inhibits budding. To test this possibility, we compared the budding of NL4-3*, which expresses Gag and Gag-Pol at their normal levels and ratio, to that of NL4-3*/TF^ter^, which expresses Gag but no Gag-Pol [15]. 293T cells were transfected with the two proviruses, cell and virus lysates were collected, and each was processed for immunoblot using antibodies specific for the Gag CA domain (**Fig. 4C**). The budding of NL4-3*/TF^ter^ was 182+/−19% (n = 3; p = 0.018) of that we observed for NL4-3*. These results demonstrate that expression of Gag-Pol inhibits HIV budding even at its normal, low level of expression [28].

## Discussion

Virus budding is a critical step in the HIV lifecycle. The prevailing paradigm of HIV budding proposes that the p6 domain of Gag plays a critical role, recruiting the ESCRT machinery to sites of virus assembly where it then catalyzes virion budding [1], [2]. This hypothesis is consistent with a wide array of empirical observations, including the severe budding defect of HIV Gag-Pol, which lacks the p6 domain. However, we show here that the severe budding defect of HIV Gag-Pol cannot be explained by the absence of p6. This conclusion is based primarily on the fact that NL4-3*/SP2^F1ter^ budded normally, even though it only expresses the first 433 amino acids of Gag (the MA, CA, SP1, and NC domains) and lacks the p6 domain. Although this observation is inconsistent with the prevailing model of the p6 domain in HIV budding, it supports the alternative hypothesis that HIV budding follows the normal, non-viral pathway of EMV biogenesis [4], [14], [15], [19], [20], [24].

Our data also demonstrate that Pol acts in *cis* to block the budding of Gag-Pol. Furthermore, the severe budding defect of PR-deficient Gag-Pol means that the Pol polypeptide itself functions as an inhibitory budding element. This is significant, as it has been previously hypothesized that the budding defect of Gag-Pol might be caused by its overexpression of PR activity [25], [26], [27]. The inhibitory effect of Pol on budding is not without precedent, as HIV has another *cis*-acting inhibitor of budding, the IBS that it located in the SP2 domain of Gag [15]. The *cis*-acting inhibitor in Pol mapped primarily to its RT domain, though the PR domain might also play a role. Regardless of its precise location, our data indicates that the inhibitory budding element in Pol also impairs the budding of HIV in *trans*. This was most obvious in our two virus expression experiments, where high levels of Gag-Pol effectively blocked HIV budding. However, Pol also had an inhibitory effect on HIV budding in the context of our control HIV provirus, where Gag-Pol expression caused an ∼2-fold reduction in HIV budding. Taken together, these data reinforce the concept that HIV budding is controlled by both positive and inhibitory budding signals, some that are shared with the non-viral EMV biogenesis pathway, and some that are unique to HIV [14], [15].

Our observations also raise the question of why HIV possesses inhibitory budding signals like the one detected here in Pol, and the IBS we identified previously in the SP2 domain of Gag [15]. Currently, we do not have a mechanistic model for why HIV would benefit from the presence of an IBS. Given the central role of budding in the HIV lifecycle, the existence of IBSs within the Gag and Gag-Pol proteins seem incongruous, and there is clearly a need for further research to identify the functional significance of these elements in the HIV lifecycle. However, it is clear that inhibitory budding signals do exist within HIV, that they can have a pronounced impact on HIV budding, and that their discovery has a significant impact on how certain empirical observations should be interpreted. In short, the discovery of inhibitory budding signals in HIV is altering our view of the *cis*-acting elements that drive HIV budding, away from the p6-dependent model of HIV budding, and towards the EMV-based model of HIV budding.

## Materials and Methods

### Cell culture and DNA transfection

293T (CRL-11268) cells were obtained from The American Type Culture Collection (Manasas, VA, USA). The growth medium in all experiments was DMEM supplemented with 10% fetal bovine serum, and growth conditions were 37°C, 5% CO_2_, and 90–100% humidity. Prior to transfection, 293T cells were released from the plate by incubation in a 0.05% tryspin/EDTA solution (Gibco/BRL, Bethesda MD, USA), pelleted by centrifugation at 200× *g*, and resuspended in growth medium at 7.5×10^5^ cells/ml. Transfection was carried out by combining DNA (10 µg total, unless otherwise stated) and 500 µls of the cell suspension in a 4 mm gap electroporation cuvette, and then electroporating the cells at 24 ohms, 300 volts, and capacitance of 800 uF, using a BTX ECM 600 electroporator (Harvard Apparatus, Holliston, MA). Following electroporation, the cells were resuspended in 10 mls of growth medium, plated on 10 cm tissue culture dishes, and grown in the incubator for an additional two days.

### Plasmids

The HIV proviruses pNL4-3-ΔE-GFP (pNL4-3*) and NL4-3*/TF^ter^ were described previously [15], [29]. To generate the mutant proviruses pNL4-3*/Gag-Pol^fuse^, pNL4-3*/Gag-Pol^fuse^/PR^D25A^, pNL4-3*/NC^F56ter^, pNL4-3*/Gag-Pol^fuse^/PR^P1ter^ and pNL4-3*/Gag-Pol^fuse^/PR^D25A^/RT^P1ter^ we first amplified the internal ApaI-SbfI fragment of pNL4-3-ΔE-GFP [29] using sets of nested primers designed to introduce the desired mutations (**Table 1**). Next, each amplified fragment was cleaved with ApaI and SbfI and inserted between the Apa I and Sbf I sites of pNL4-3-ΔE-GFP. The pNL4-3*/Gag-Pol^fuse^/PR^D25A^/p15^Y1ter^ and pNL4-3*/Gag-Pol^fuse^/PR^D25A^/IN^F1ter^ proviruses were created by amplifying the internal AgeI-EcoRI fragment from pNL4-3-ΔE-GFP [29] using nested sets of primers designed to introduce the desired mutations, followed by cleaving the amplified products with AgeI and EcoRI and inserting them into the AgeI and EcoRI sites of pNL4-3*/Gag-Pol^fuse^/PR^D25A^. All amplified regions of the plasmids were sequenced, and experiments were only performed using clones possessing the desired sequence. The relevant mutations in these proviruses are shown in a limited sequence alignment (**Table 1**).

**Table 1:** Primers used.

primer name	primer sequence
FGag-Pol-ApaI	5′-CAAAAATTGCAGGGCCCCTAGG-3′
RGag-Pol-SbfI	5′- TTTAACCCTGCAGGATGTGG-3′
RpGag-Pol	5′- AGATCTTCCCTTAAAAAATTAGCCTGTCTC-3′
FpGag-Pol	5′- GCTAATTTTTTAAGGGAAGATCTGGCC-3′
RpNL-TF-F1T	5′- CTTCCCTAATTAATTAGCCTGTCTCTCAGTACAATC -3′
FpNL-TF-F1T	5′- GAGACAGGCTAATTAATTAGGGAAGATCTGG-3′
RpNL-PR-P1T	5′- CACCTGCAGGTTAGAAGCTAAAGGATACAGTTCCTTG-3′
RpNL-RT-P1T	5′- CACCTGCAGGTTAAAAATTTAAAGTGCAGCCAATCTG-3′
FGag-Pol-AgeI	5′- GATTCTAAAAGAACCGGTACATG-3′
RGag-Pol-EcoRI	5′- CAGTTGTTGCAGAATTCTTATTATG-3′
RpNL-p15-Y1T	5′- CTTTTCCATGTGTTAGAAAGTTTCTGCTCCTATTATGG-3′
FpNL-p15-Y1T	5′- CAGAAACTTTCTAACACATGGAAAAGATTAGTAAAAC-3′
RpNL-IN-F1T	5′- CTTTTCCATGTGTTATAGTACTTTCCTGATTCCAGCAC-3′
FpNL-IN-F1T	5′- GGAAAGTACTATAACACATGGAAAAGATTAGTAAAAC -3′

### Virus preparations, antibodies, and immunoblot

Cell and virus lysates were performed essentially as described [15]. In brief, 293T cells were incubated for two days, followed by collection of the tissue culture supernatant, and washing of cells once in DMEM. Cells were then lysed by the addition of SDS-PAGE sample buffer. To isolate HIV particles, the tissue culture supernatant was first subjected to centrifugation at 5,000× *g* for 15 minutes. The pellet was discarded and the resulting supernatant was passed through a sterile, 0.22 micrometer filter. The filtered supernatant was then spun at 10,000× *g* for 30 minutes, the pellet was discarded, and the supernatant was again spun at 10,000× g for 30 minutes. The pellet was discarded and the resulting supernatant was spun at 70,000× g for 60 minutes to pellet HIV particles. The supernatant was discarded and the virus-containing pellet was lysed by resuspension in SDS-PAGE sample buffer.

For immunoblot experiments, cell and virus lysates (in all cases, a 1∶20 ratio of cell and virus lysates) were separated by SDS-PAGE, transferred to PVDF membranes, and processed for immunoblot using specific primary antibodies to HIV Gag CA domain (mouse anti-Gag monoclonal antibody (3537), AIDS Research & Reference Reagent Program (NIAID, NIH) and HRP-linked secondary antibodies (Jackson Immunoresearch, West Grove, PA, USA)). Gag CA-containing proteins were detected by chemiluminescent exposure of X-ray film and developed films were digitally scanned and converted to TIFF files using Adobe Photoshop CS2 and all images were assembled in Adobe Illustrator CS2. Protein band intensities were determined using ImageJ software and these data were used to determine the budding efficiency (vesicle-associated signal/(vesicle-associated + cell-associated signals)) of each virus. Relative budding was determined by comparing the budding of each test virus to that of a control, which was assigned an arbitrary value of 1. The statistical analysis of relative budding included the calculation of averages, standard deviations, and the calculation of p values (Student's *t* test).

## References

[1] Bieniasz PD (2009). The cell biology of HIV-1 virion genesis.. Cell Host Microbe.

[2] Morita E, Sundquist WI (2004). Retrovirus budding.. Annu Rev Cell Dev Biol.

[3] Freed EO, Martin MO, Knipe DM, Howley PM (2006). HIVs and their replication.. Fields Virology. fifth ed.

[4] Gould SJ, Booth AM, Hildreth JE (2003). The Trojan exosome hypothesis.. Proc Natl Acad Sci U S A.

[5] Hurley JH, Boura E, Carlson LA, Rozycki B (2010). Membrane budding.. Cell.

[6] Gottlinger HG, Dorfman T, Sodroski JG, Haseltine WA (1991). Effect of mutations affecting the p6 gag protein on human immunodeficiency virus particle release.. Proc Natl Acad Sci U S A.

[7] Huang M, Orenstein JM, Martin MA, Freed EO (1995). p6Gag is required for particle production from full-length human immunodeficiency virus type 1 molecular clones expressing protease.. J Virol.

[8] Garrus JE, von Schwedler UK, Pornillos OW, Morham SG, Zavitz KH (2001). Tsg101 and the vacuolar protein sorting pathway are essential for HIV-1 budding.. Cell.

[9] Strack B, Calistri A, Craig S, Popova E, Gottlinger HG (2003). AIP1/ALIX is a binding partner for HIV-1 p6 and EIAV p9 functioning in virus budding.. Cell.

[10] von Schwedler UK, Stuchell M, Muller B, Ward DM, Chung HY (2003). The protein network of HIV budding.. Cell.

[11] Raiborg C, Stenmark H (2009). The ESCRT machinery in endosomal sorting of ubiquitylated membrane proteins.. Nature.

[12] Hurley JH (2008). ESCRT complexes and the biogenesis of multivesicular bodies.. Curr Opin Cell Biol.

[13] Demirov DG, Orenstein JM, Freed EO (2002). The late domain of human immunodeficiency virus type 1 p6 promotes virus release in a cell type-dependent manner.. J Virol.

[14] Fang Y, Wu N, Gan X, Yan W, Morrell JC (2007). Higher-order oligomerization targets plasma membrane proteins and HIV gag to exosomes.. PLoS Biol.

[15] Gan X, Gould SJ (2011). Identification of an inhibitory budding signal that blocks the release of HIV particles and exosome/microvesicle proteins.. Mol Biol Cell.

[16] Simons M, Raposo G (2009). Exosomes–vesicular carriers for intercellular communication.. Curr Opin Cell Biol.

[17] Thery C, Ostrowski M, Segura E (2009). Membrane vesicles as conveyors of immune responses.. Nat Rev Immunol.

[18] Nguyen DG, Booth A, Gould SJ, Hildreth JE (2003). Evidence that HIV budding in primary macrophages occurs through the exosome release pathway.. J Biol Chem.

[19] Krishnamoorthy L, Bess JW, Preston AB, Nagashima K, Mahal LK (2009). HIV-1 and microvesicles from T cells share a common glycome, arguing for a common origin.. Nat Chem Biol.

[20] Booth AM, Fang Y, Fallon JK, Yang JM, Hildreth JE (2006). Exosomes and HIV Gag bud from endosome-like domains of the T cell plasma membrane.. J Cell Biol.

[21] Marsh M, Theusner K, Pelchen-Matthews A (2009). HIV assembly and budding in macrophages.. Biochem Soc Trans.

[22] Shen B, Wu N, Yang JM, Gould SJ (2011). Protein targeting to exosomes/microvesicles by plasma membrane anchors.. J Biol Chem.

[23] Llewellyn GN, Hogue IB, Grover JR, Ono A (2010). Nucleocapsid promotes localization of HIV-1 gag to uropods that participate in virological synapses between T cells.. PLoS Pathog.

[24] Shen B, Fang Y, Wu N, Gould SJ (2011). Biogenesis of the posterior pole is mediated by the exosome/microvescile protein-sorting pathway.. Journal of Biological Chemistry.

[25] Park J, Morrow CD (1991). Overexpression of the gag-pol precursor from human immunodeficiency virus type 1 proviral genomes results in efficient proteolytic processing in the absence of virion production.. J Virol.

[26] Smith AJ, Srinivasakumar N, Hammarskjold ML, Rekosh D (1993). Requirements for incorporation of Pr160gag-pol from human immunodeficiency virus type 1 into virus-like particles.. J Virol.

[27] Karacostas V, Wolffe EJ, Nagashima K, Gonda MA, Moss B (1993). Overexpression of the HIV-1 gag-pol polyprotein results in intracellular activation of HIV-1 protease and inhibition of assembly and budding of virus-like particles.. Virology.

[28] Jacks T, Power MD, Masiarz FR, Luciw PA, Barr PJ (1988). Characterization of ribosomal frameshifting in HIV-1 gag-pol expression.. Nature.

[29] Zhang H, Zhou Y, Alcock C, Kiefer T, Monie D (2004). Novel single-cell-level phenotypic assay for residual drug susceptibility and reduced replication capacity of drug-resistant human immunodeficiency virus type 1.. J Virol.

